# Detection of Avian Influenza H5–Specific Antibodies by Chemiluminescent Assays

**DOI:** 10.3201/eid3201.251117

**Published:** 2026-01

**Authors:** Ana Citlali Márquez, Saina Beitari, Tahereh Valadbeigy, Linda Hoang, Yohannes Berhane, Agatha N. Jassem

**Affiliations:** British Columbia Centre for Disease Control, Vancouver, British Columbia, Canada (A.C. Márquez, S. Beitari, T. Valadbeigy, L. Hoang, A.N. Jassem); University of British Columbia, Vancouver (A.C. Márquez, L. Hoang, A.N. Jassem); Canadian Food Inspection Agency, Winnipeg, Manitoba, Canada (Y. Berhane); University of Manitoba, Winnipeg (Y. Berhane); University of Saskatchewan, Saskatoon, Saskatchewan, Canada (Y. Berhane)

**Keywords:** influenza, H5N1, immunoassays, serology, avian influenza, viruses, Canada

## Abstract

We evaluated 2 electrochemiluminescence serologic assays to detect avian influenza H5 antibodies. The assays identified H5 antibodies from both serum and dried blood spots and had strong specificity and minimal cross-reactivity in human and avian samples. Such assays can support populationwide serologic surveys aimed at assessing population-level immunity.

Since late 2021, highly pathogenic avian influenza virus (HPAIV) epizootic events in poultry have been reported worldwide, driven by HPAIV A(H5N1) clade 2.3.4.4b (*1*). Although wild birds are the natural reservoir for avian influenza viruses, H5N1 clade 2.3.4.4 has also infected wild mammals and, more recently, dairy cattle, raising concerns about viral adaptation to mammalian hosts, cross-species transmission, and increased risk for zoonotic spillover (*2*). Infections among agricultural workers have further amplified concern about possible human-to-human transmission and broader public health implications (*3*,*4*). Monitoring population-level exposure is critical for assessing susceptibility of human populations to H5N1 clade 2.3.4.4b infection and informing appropriate risk mitigation strategies.

Determination of HPAIV prevalence relies on passive surveillance from testing symptomatic persons through PCR and sequencing to detect active infections (*5*). However, HPAIV H5N1 can manifest as a mild infection and be underdetected and underreported (*6*). Serologic tools that detect virus-specific antibodies indicating prior exposures or infection can provide a more accurate estimate of infection prevalence at the population level. Serologic testing for HPAIV H5Nx subtype-specific antibodies has largely relied on hemagglutinin inhibition (HAI), neuraminidase inhibition, and microneutralization (MN) assays (*7*). HAI and MN are considered reference methods because of their high specificity and sensitivity and are often used in a complementary manner for influenza antibody characterization and quantitation. However, those assays are time-consuming and must be conducted primarily in a Biosafety Level 3 laboratory when performed with H5Nx viruses (*7*). Interpretation of such assays can be challenging, leading to undesired variability and lower sensitivity. We evaluated 2 assays from Meso Scale Discovery (MSD, https://www.mesoscale.com) for the detection of H5 antibodies to determine their feasibility and scalability for serosurveys.

## The Study

The first of the 2 assays is a multiplex panel, the V-PLEX Respiratory Panel 7 (IgG) Kit, which includes antigens for SARS-CoV-2, respiratory syncytial virus, seasonal influenza, and avian influenza H5 (flu A/Ghana/39/2021 H5 clade 2.3.4.4b). The assay is designed to work only with human samples. The second assay, the Influenza H5 Bridging Serology Kit, uses a biotinylated H5 protein designed to detect H5 antibodies from multiple species, this antigen is the head fragment from the hemagglutinin of A/Ghana/AVL-763_21VIR750–39/2021 (H5N1 clade 2.3.4.4b). The second assay also includes an H1 cross-reactivity blocker to minimize nonspecific detection of H1 antibodies in human samples. We evaluated the assays only for influenza, not other respiratory pathogens.

For testing, we used residual serum samples previously submitted for clinical testing to the British Columbia Centre for Disease Control (Vancouver, BC, Canada). Positive samples (n = 6) were obtained from sequential serum samples collected 12–22 days after onset from a patient infected with H5N1 clade 2.3.4.4b virus (*8*). Those serum samples were previously shown to be reactive for -H5 by HAI and MN assays at the National Microbiology Laboratory (Winnipeg, MN, Canada) (*9*). We also tested samples collected from close contacts (n = 8) of the infected patient that were determined to be negative for H5N1 by HAI and MN at the National Microbiology Laboratory. We used 23 residual antenatal serum samples submitted to the British Columbia Centre for Disease Control in 2022 for routine clinical testing as negative controls. To assess assay cross-reactivity, we tested residual samples from persons infected during the 2024–25 respiratory season with influenza A (H1, n = 15; H3, n = 6), confirmed by PCR.

In the multiplex assay (Figure, panel A), samples from the persons infected with H5N1 demonstrated the highest geometric mean concentration (GMC) to the H5 antigen (GMC = 630,877 arbitrary units [AU]/mL, 95% CI 622,836–639,021 AU/mL). Antenatal samples, H5N1 contact samples, and samples from those infected with H3 demonstrated low reactivity compared with samples from the infected patient (GMC <8,650 AU/mL). Samples from persons recently infected with influenza H1 exhibited some reactivity to the H5 antigen, suggesting cross-reactivity between H1 and H5 antibodies; however, the signal from H1-infected samples was significantly lower (GMC = 16,977 AU/mL, 95% CI = 4,412–65,320 AU/mL) than that of the confirmed H5N1 case (p<0.0001). Despite strong subtype-specific responses in H1- and H3-infected samples, the H5-infected samples demonstrated even higher signal intensity to both H1 and H3 antigens, suggesting the presence of antibodies to both H1 and H3 HA from past exposures (Appendix Figure). Cross-reactivity between H5 and H1 antibodies is expected because of the recognition of shared, conserved epitopes in the hemagglutinin stalk region. However, this cross-reactivity could only be assessed if acute and convalescent samples were available; in this case, only convalescent samples from the H5-infected patient were available (*10*).

**Figure:**
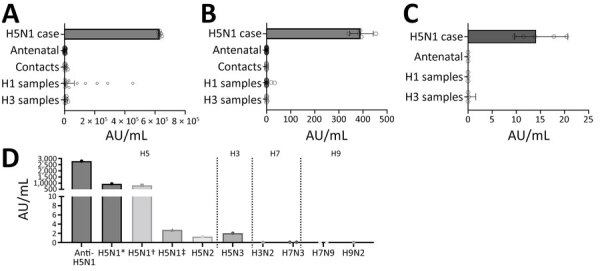
Specific detection of H5 antibodies in human and avian samples in a study on detection of avian influenza H5–specific antibodies by chemiluminescent assays. A) Results of human serum sample testing by using the MSD V-Plex Respiratory Panel 7 (IgG) kit (MSD, https://www.mesoscale.com), diluted 1:5,000 in the manufacturer-provided diluent. B) Results of human serum sample testing by using the MSD Influenza H5 Bridging Serology Assay (MSD, https://www.mesoscale.com), diluted 1:20 and prepared according to the manufacturer’s instructions. C) Results of dried blood spot sample testing by using the MSD Influenza H5 Bridging Serology Assay at 1:10 dilution. D) H5 antibody polyclonal and avian sample results obtained from chickens inoculated with various influenza subtypes and tested on the MSD Influenza H5 Bridging Serology Assay according to the manufacturer's instructions. Antibody titer production was confirmed by hemagglutinin inhibition assay. *A/chicken/Vietnam/14/2005; †H5 DNA vaccinated with HA from A/mallard/BC/373/2005 (H5N2) and challenged with A/chicken/Vietnam/14/2005 (H5N1); ‡A/teal/Germany/Wv632/2005; AU, arbitrary units; MSD, Meso Scale Discovery.

The bridging serology assay functions on the basis of a biotinylated H5 antigen that binds to a streptavidin-coated plate, along with an H5-conjugated detection antigen. This setup forms a bridging complex in the presence of H5 antibodies, enabling their specific detection across species. We tested the same set of samples by using this assay (Figure, panel B). Unlike the multiplex assay, background reactivity observed in H1-infected samples was largely eliminated (GMC = 0.18 AU/mL, 95% CI = 0.0066–4.88 AU/mL), and the assay specifically detected antibodies only in the samples from the H5N1-infected persons (GMC = 390.5 AU/mL, 95%CI = 344.5–442.7 AU/mL).

To assess the suitability of the assay across different sample types, we prepared contrived dried blood spot (DBS) samples by mixing packed red blood cells with either serum from antenatal testing or H5N1 cases. DBS samples from the H5N1 cases had detectable H5 antibodies, although the signal was lower than that observed in serum samples (GMC = 14.12 AU/mL, 95% CI = 9.627–20.7 AU/mL). We detected no cross-reactive background in the rest of the sample groups (Figure, panel C).

To evaluate the cross-species applicability of the assay, we tested a commercially available chicken H5 polyclonal antibody (Creative Diagnostics, https://www.creative-diagnostics.com) along with serum samples from chickens that were experimentally infected with low pathogenicity or inactivated highly pathogenic H5 influenza viruses, vaccinated with an H5N1 DNA vaccine, and subsequently challenged with inactivated H5N1 or exposed to other HA subtypes (Table). H5 antibodies were detectable in samples from animals infected with H5 viruses; the highest levels were observed in a vaccinated animal with H5 DNA vaccine and challenged with H5N1 (A/chicken/Vietnam/14/2005) and in a chicken infected with H5N1 (A/chicken/Vietnam/14/2005) (Figure, panel D). We observed no cross-reactivity in serum from animals infected with non-H5 subtypes.

**Table:** Viruses used to inoculate avian species for serum collection in a study on detection of avian influenza H5-specific antibodies by chemiluminescent assays*

Virus	Name	Lineage	Host	HAI titer†
Anti-H5N1	Anti-H5N1 polyclonal antibody‡	Clade 2.3.4.4b	Chicken	N/A
H5N1	A/chicken/Vietnam/14/2005	Clade 2.3.2	Chicken	256
H5N1	H5 DNA vaccinated with HA from A/mallard/BC/373/2005 (H5N2) and challenged with A/chicken/Vietnam/14/2005 (H5N1)	Vaccine: North American; challenge: Clade 2.3.2	Duck	1,024
H5N1	A/teal/Germany/Wv632/2005	Eurasian LPAI	Chicken	256
H5N2	A/turkey/Minn/3689-1551-1981	North American	Chicken	128
H3N2	A/DK/ON/05/2000	Triple reassortant North American	Chicken	128
H5N3	A/TY/CA/35621/1984	North American	Chicken	128
H7N3	A/chicken/BC/CN-7/2004	North American	Chicken	256
H7N9	A/Anhui/001/2013	East Asian	Chicken	128
H9N2	A/Ty/Mn/12877/1285/1981	North American	Chicken	128

*HA, hemagglutinin; HAI, hemagglutinin inhibition. †Experimental serum generated at the National Centre for Foreign Animal Disease, Canadian Food Inspection Agency, where HAI testing was also performed. ‡Creative Diagnostics, https://www.creative-diagnostics.com.

## Conclusions

Our results show that the V-PLEX Respiratory Panel 7 (IgG) and the H5 Bridging Serology serologic assays can specifically detect H5 antibodies in humans. In the V-PLEX panel, serum samples from H1 infected patients showed varied levels of cross-reactivity to the H5 antigen, whereas samples from H3 infected patients did not show cross-reactivity to the H5 antigen. The serology bridging assay shows a marked reduction in cross-reactivity with closely related hemagglutinin subtypes, such as H1 in humans. This assay is compatible with DBS and can detect antibodies from avian species.

This study is limited by the small number of H5N1-positive human samples available for testing and the absence of manufacturer-defined reactivity cutoffs. Additional samples from confirmed H5-infected patients with mild or moderate symptoms would enable determination of thresholds to distinguish between positive and negative results. For avian samples, the main limitation is that serum was obtained from experimentally inoculated animals; thus, the assay’s performance in wild birds, or in other species, remains to be evaluated.

Although HAI and MN assays remain the reference methods for influenza subtyping, the assays we describe provide a versatile and scalable alternative. The assays have strong potential as screening tools for detecting anti-H5 in both humans and animals and for use in serologic surveys or contact tracing.

Appendix Additional information about detection of avian influenza H5–specific antibodies by chemiluminescent assays.
