# Correction: Vol. 32, No. 1

**DOI:** 10.3201/eid3203.C13203

**Published:** 2026-03

**Keywords:** errata, erratum, correction

Funding information was missing for Detection of Avian Influenza H5–Specific Antibodies by Chemiluminescent Assays (A.C. Márquez et al.). The article has been corrected online (https://wwwnc.cdc.gov/eid/article/32/1/25-1117_article).

