# Supplementary material for: Detection of Avian Influenza H5–Specific Antibodies by Chemiluminescent Assays
Source: Emerg Infect Dis. 2026 Jan;32(1):129–32. doi: 10.3201/eid3201.251117 (PMC12870130; doi:10.3201/eid3201.251117)
Supplement: Appendix — Additional information about detection of avian influenza H5–specific antibodies by chemiluminescent assays. [file 25-1117-Techapp-s1.pdf]

Article DOI: https://doi.org/10.3201/eid3201.251117
EID cannot ensure accessibility for supplementary materials supplied by authors. Readers who have difficulty accessing supplementary content should contact the authors for assistance.

Detection of Avian Influenza H5–Specific Antibodies by Chemiluminescent Assays

Appendix

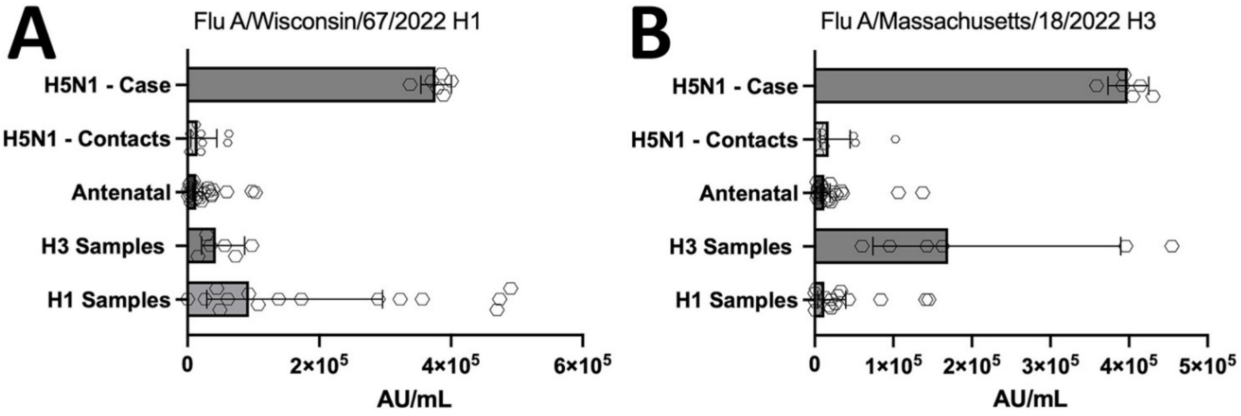

Appendix Figure. Antibodies in human samples from an individual infected with H5N1 shows cross-reactivity with H3 and H1 antigens. A) Human serum samples were tested using the MSD V-Plex Respiratory Panel 7 (IgG) kit, diluted 1:5000 in the manufacturer-provided diluent.
